# Prevalence of hyperlipidemia in Shanxi Province, China and application of Bayesian networks to analyse its related factors

**DOI:** 10.1038/s41598-018-22167-2

**Published:** 2018-02-28

**Authors:** Jinhua Pan, Zeping Ren, Wenhan Li, Zhen Wei, Huaxiang Rao, Hao Ren, Zhuang Zhang, Weimei Song, Yuling He, Chenglian Li, Xiaojuan Yang, LiMin Chen, Lixia Qiu

**Affiliations:** 1 Department of Health Statistics, School of Public Health, Shanxi Medical University, No. 5 6 XinJian South Road, Taiyuan, Shanxi 030001 China; 2 Shanxi Centre for Disease Control and Prevention, Taiyuan, Shanxi 030012 China; 3 Institute for Communicable Disease Control and Prevention, Qinghai Provincial Center for Disease Control and Prevention, Xining, Qinghai 810007 China

## Abstract

This study aimed to obtain the prevalence of hyperlipidemia and its related factors in Shanxi Province, China using multivariate logistic regression analysis and tabu search-based Bayesian networks (BNs). A multi-stage stratified random sampling method was adopted to obtain samples among the general population aged 18 years or above. The prevalence of hyperlipidemia in Shanxi Province was 42.6%. Multivariate logistic regression analysis indicated that gender, age, region, occupation, vegetable intake level, physical activity, body mass index, central obesity, hypertension, and diabetes mellitus are associated with hyperlipidemia. BNs were used to find connections between those related factors and hyperlipidemia, which were established by a complex network structure. The results showed that BNs can not only be used to find out the correlative factors of hyperlipidemia but also to analyse how these factors affect hyperlipidemia and their interrelationships, which is consistent with practical theory, is superior to logistic regression and has better application prospects.

## Introduction

Cardiovascular disease (CVD) is the leading cause of death worldwide, accounting for 30% of all deaths^1^. As an influencing factor of cardiovascular disease^2–5^, hyperlipidemia plays an important role in the occurrence and development of CVD^6^. However, with increased economic development, improvement of living standards, and changes in lifestyle, the prevalence of hyperlipidemia has been gradually increasing in China^1,7–9^. The results of the 2002 Chinese National Nutrition and Health Survey showed that the prevalence of hyperlipidemia was 18.6%^1^. In 2007, a survey of 43,368 residents in China over age 18 years demonstrated that the prevalence of hyperlipidemia had increased to 33.97%^7^. Another report in 2014 showed that the prevalence of hyperlipidemia in China reached as high as 41.9%^9^. Hyperlipidemia has become an important public health problem; therefore, it is of great importance to comprehensively analyse the related factors of hyperlipidemia to prevent its occurrence.

As shown in previous studies, gender, age, lifestyle, obesity, diabetes mellitus, dietary structure, and other factors directly or indirectly affect the incidence and progress of hyperlipidemia^6,7,10,11^. Most previous studies on factors related to hyperlipidemia have used logistic regression based on independent variables, and odds ratio values to reflect the degree of association; however, in reality, these factors are often interdependent and may have a complex network structure, which cannot meet the assumptions of a logistic regression model and lead to the failure of logistic regression to describe this relationship. In addition, the relating factors of logistic regression are parallel, which cannot infer the part they play in the occurrence and development of hyperlipidemia. Mancini *et al*.^12,13^ stated that traditional statistical methods such as logistic regression, are ineffective for describing the relationship between variables in the biomedical domain because of their limitations of independency. Bayesian networks (BNs) can overcome this shortcoming and has become a popular method for analysing the relationship between variables in biomedical field. The BN method is a technique based on the probability of uncertainty reasoning and has no strict requirements for statistical assumptions. In the BN method, a directed acyclic graph (DAG) is constructed to intuitively reflect the potential relationship between factors, and a conditional probability distribution table is used to reflect the strength of association^14^. Unlike logistic regression, BNs allow for estimating the subsequent probability of any target variable given any set of conditioning variables^15,16^, which can predict the probability of having dyslipidemia in a more flexible manner. Tabu search is a metaheuristic approach proposed by Glover and it is one of the most efficient optimization techniques that incorporates adaptive memory to escape local search and find the global optimum^17^. Hence, we applied BNs optimized with a tabu search algorithm to jointly model dyslipidemia and its related factors and determine how these factors impact dyslipidemia, to offer comprehensive strategies for effectively reducing the incidence of hyperlipidemia.

## Results

### Characteristics of the study population

Among the 4,776 initial participants in our study, we excluded 671 with incomplete data. Finally, A total of 4,105 participants (1,748 men and 2,357 women) were included in this study; 2,581 (62.9%) participants came from rural regions and 1,524 (37.1%) were from urban areas. The median age was 51.5 years, ranging from age 18 to 108 years.

### Detection rate of dyslipidemia

**Figure 1:**
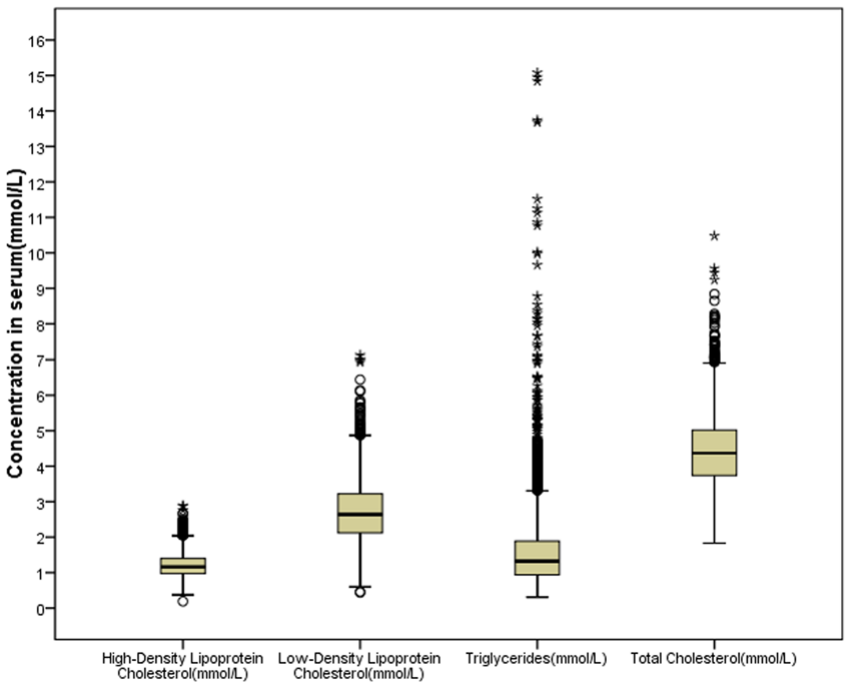
Concentrations of total cholesterol, triglycerides, high-density lipoprotein cholesterol, and low-density lipoprotein cholesterol in blood samples among study participants. The figure was plotted using IBM SPSS 22.0 (https://www.ibm.com).

Figure 1 shows the distribution of TC, TG, HDL-C, and LDL-C in participant blood samples. Of 4,105 participants, 1,748 were diagnosed as having dyslipidemia, The detection rate was 42.6% (45.7% for male, 40.3% for female). The detection rates of hypercholesterolemia, hypertriglyceridemia, HDL-C, and LDL-C were 6.1%, 16.4%, 33.8%, and 5.1%, respectively. The main types of hyperlipidemia in Shanxi Province were low HDL-C, followed by hypertriglyceridemia.

### Univariate analysis

**Figure 2:**
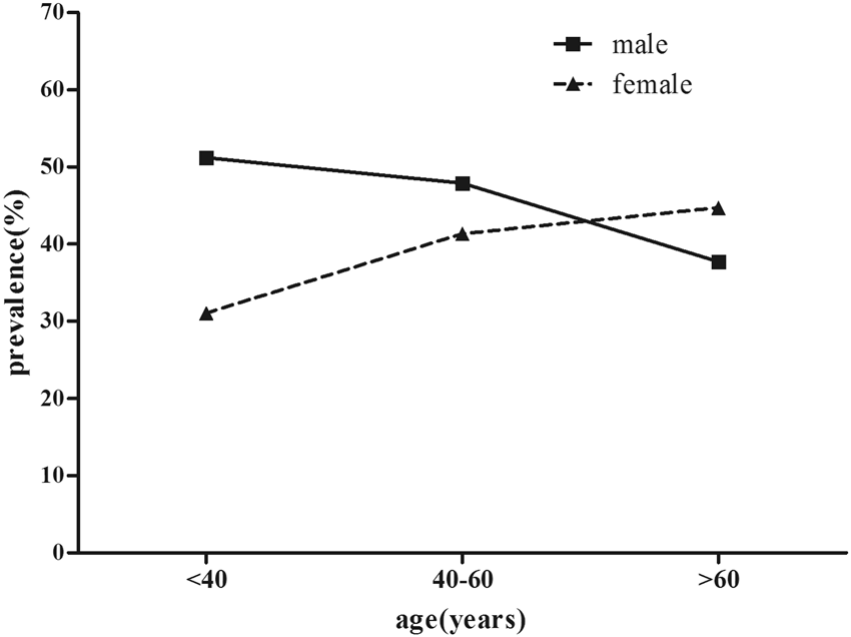
Prevalence of dyslipidemia by age groups. The figure was plotted using GraphPad Prism 5.01 (https://www.graphpad.com/).

Supplementary Table S1 shows related factors and their assignment. Supplementary Tables S2–S5 show differences in the prevalence of dyslipidemia among participants with different characteristics. Factors such as old age, male, rural residence, employers, higher educational level, higher BMI value, sufficient intake of vegetables, insufficient physical activity, central obesity, and having a history of hypertension and diabetes mellitus showed a higher prevalence of dyslipidemia (all, P < 0.10). As depicted in Fig. 2 and Table 1, men younger than age 60 years were more inclined to have higher prevalence of dyslipidemia than women in the same age group (P < 0.1), whereas the detection rate of dyslipidemia increased with age in women (P < 0.1); women over age 60 years had a significantly higher detection rate than men in the same age group (P < 0.1).

**Table 1:** Detection rate of dyslipidemia by different age groups and gender.

Age (years)	Gender	Cases	hyperlipidemia	Prevalence (%)	*χ* ^2^	*P*
<40	Male	326	167	51.2	31.263	<0.001
	Female	422	131	31.0		
40~	Male	934	447	47.9	9.595	0.002
	Female	1367	565	41.3		
60~	Male	488	184	37.7	5.319	0.021
	Female	568	254	44.7		

### Multivariate analysis

We conducted a multivariate logistic regression analysis using stepwise method (α_*in*_ = 0.10, α_*out*_ = 0.20) to select variables, with the presence of dyslipidemia as the dependent variable; independent variables were those that were significantly associated with dyslipidemia in univariate analysis. The multivariate analysis revealed that dyslipidemia was significantly associated with gender (OR 0.716, 95% CI: 0.628–0.817), age (OR 0.906, 95% CI: 0.817–1.005), region (OR 0.875, 95% CI: 0.762–1.005), occupation as a farmer (OR 0.844, 95% CI: 0.725–0.982), vegetable intake level (OR 1.080, 95% CI: 0.992–1.177), sufficient physical activity (OR 0.785, 95% CI: 0.653–0.944), BMI (OR 1.469, 95% CI: 1.327–1.626), central obesity (OR 1.698, 95% CI: 1.433–2.011), hypertension (OR 1.276, 95% CI: 1.109–1.468), and diabetes mellitus (OR 1.318, 95% CI: 1.057–1.643) (Table 2). Central obesity was strongly associated with hyperlipidemia (OR 1.760, 95% CI: 1.491–2.078), followed by BMI (OR 1.529, 95% CI: 1.382–1.692).

**Table 2:** Multivariate logistic regression analyses on relaing factors of dyslipidemia.

Factors	*β*	SE	wald χ^2^	*P*	*OR*(95%*CI*)
Gender	−0.334	0.067	24.490	<0.001	0.716(0.628,0.817)
Age	−0.098	0.053	3.489	0.062	0.906(0.817,1.005)
Region	−0.133	0.070	3.574	0.059	0.875(0.762,1.005)
vegetable intake level	0.077	0.044	3.126	0.077	1.080(0.992,1.177)
hypertension	0.244	0.072	11.589	0.001	1.276(1.109,1.468)
diabetes mellitus	0.276	0.112	6.025	0.014	1.318(1.057,1.643)
BMI(kg/m^2^)	0.385	0.052	54.841	<0.001	1.469(1.327,1.626)
Central obesity	0.529	0.086	37.442	<0.001	1.698(1.433,2.011)
insufficient physical activity			7.741	0.021	
Normal physical activity	−0.048	0.080	0.359	0.549	0.953(0.814,1.116)
sufficient physical activity	−0.242	0.094	6.654	0.010	0.785(0.653,0.944)
Other			5.843	0.119	
Farmer	−0.170	0.077	4.828	0.028	0.844(0.725,0.982)
Retirees or unemployers	−0.099	0.137	0.520	0.471	0.906(0.693,1.185)
Employers	−0.013	0.105	0.016	0.899	0.987(0.804,1.212)
constant	−0.853	0.162	27.918	<0.001	0.426

### Bayesian networks model

**Figure 3:**
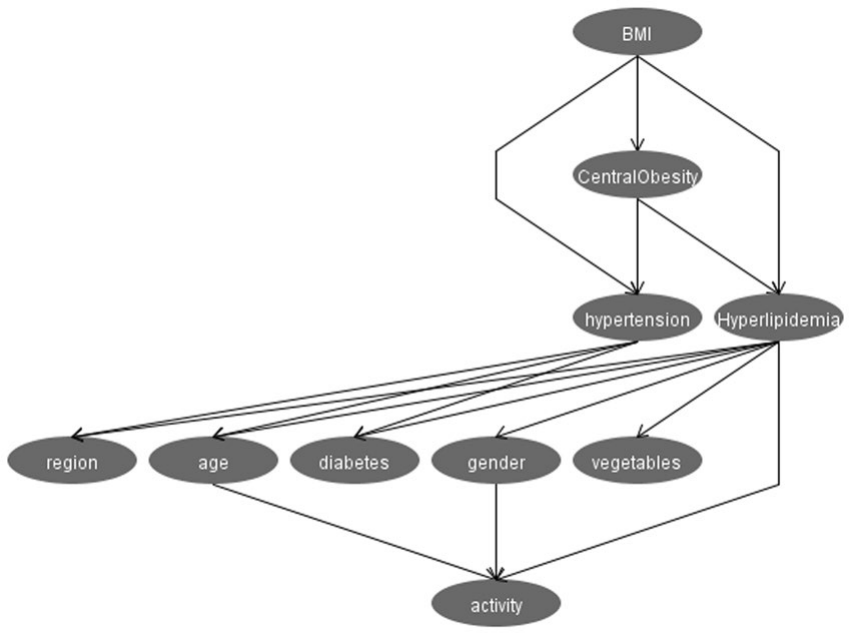
Bayesian Network model of factors relating to dyslipidemia. The figure was plotted using Weka 3.8.0 (https://www.cs.waikato.ac.nz/ml/weka/).

A probabilistic model with 10 nodes and 16 directed edges was built using BNs, considering those variables with significant differences in the multivariate logistic regression analysis (Fig. 3). Directed edges represent probabilistic dependencies between the nodes that are connected rather than the causal relationship between hyperlipidemia and the factors. Figure 3 shows that connections between hyperlipidemia and its related factors were established by a complex network structure, in which a direct connection between gender, age, region, occupation, vegetable intake level, physical activity, BMI, central obesity, diabetes mellitus, and hyperlipidemia were found (Fig. 3); in addition, hypertension was indirectly linked to hyperlipidemia through diabetes mellitus. We can also figure out the interrelationship between the related factors of hyperlipidemia from Fig. 3. For example, physical activity is related to gender and age; hypertension is associated with region, age, BMI, central obesity, and diabetes; BMI is also associated with central obesity. Supplementary Table S6 shows the CPT of hyperlipidemia, which quantitatively describes the relationship between the hyperlipidemia node and its parent nodes.

### Reasoning model

**Figure 4:**
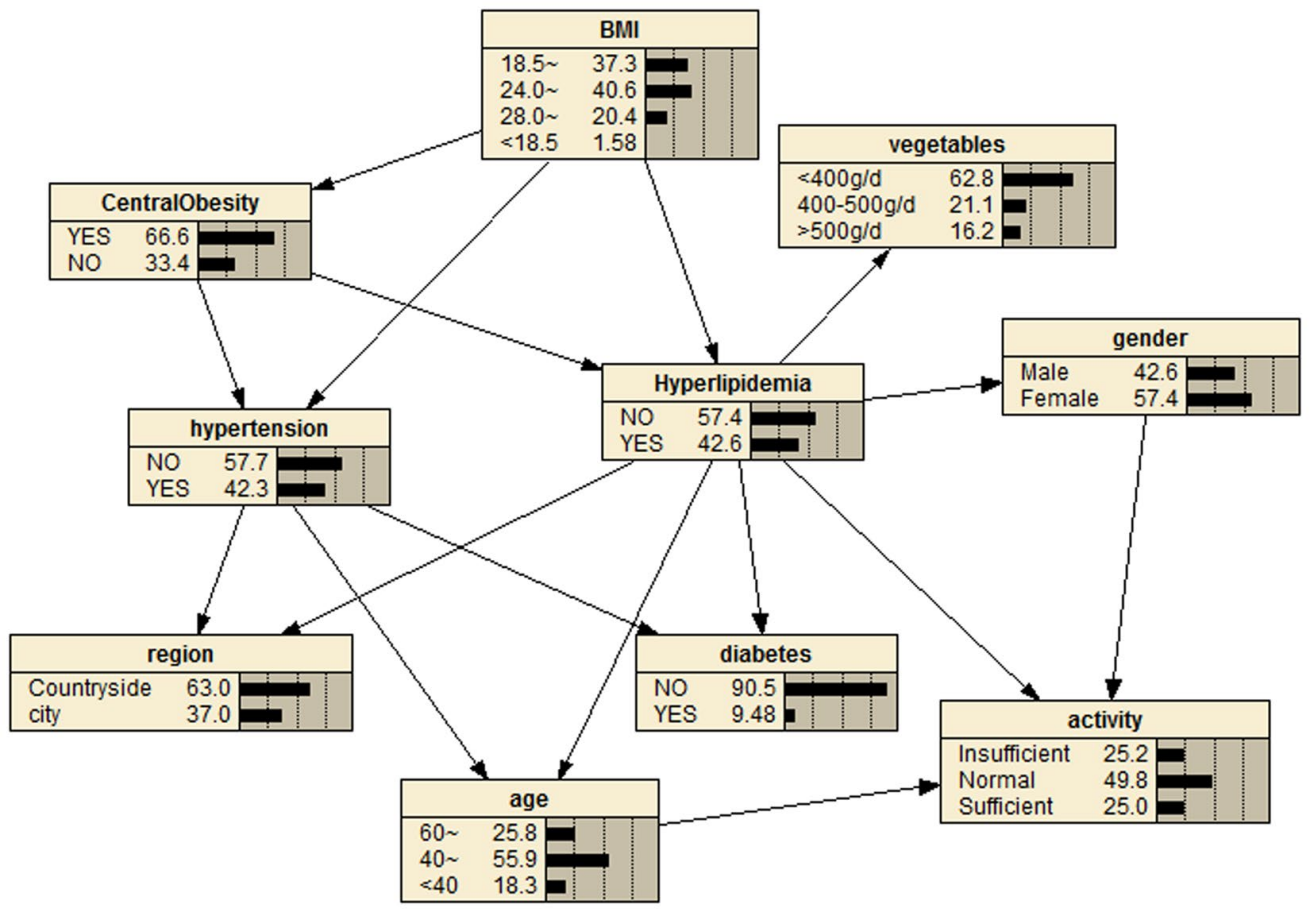
Bayesian network model. Marginal probabilities. The figure was plotted using Netica (www.norsys.com).

Marginal probabilities of the variables are shown in Fig. 4. It can be seen that the marginal probability of hyperlipidemia was 42.6%. The resulting probabilistic model can be used to quantitatively analyse the impact of these factors on hyperlipidemia by computing the conditional probabilities *P*(*y*|*xi*). For example, Supplementary Fig. S1 shows that if a person has insufficient exercise, the detection rate of hyperlipidemia increases from the marginal value of 42.6% to *P(hyperlipidemia|Lack of exercise)* = 45.1%, and the detection rate of hyperlipidemia reaches 55.2% if this person has central obesity (Supplementary Fig. S2). We can see from Supplementary Fig. S3 that if this person is also obese (according to BMI), then they have a 59.4% probability of having hyperlipidemia; the probability increases to 65.0% when this person has concurrent diabetes mellitus (Supplementary Fig. S4). Bayesian networks can also be used to study the interrelationship between related factors. For example, we can see from Supplementary Figs S5 and S6 that if a person has diabetes mellitus, the probability of developing hypertension increases to 60%, and if he has hypertension, the probability of having diabetes mellitus increases to 13.4%. In addition, if a person has insufficient exercise, the probability of having central obesity, diabetes mellitus, and hypertension is increased to 67.6%, 9.73%, and 44.9%, respectively.

## Discussion

The increasing prevalence of dyslipidemia had become a worldwide public health problem. We found a detection rate of dyslipidemia was 42.6% in Shanxi Province of China, which is considerably higher than the nationally reported prevalence of dyslipidemia^9^ (41.9%) as well as those reported in other provinces of China^1,3,11,18^. Therefore, Shanxi Province should give more attention to the prevention and control of hyperlipidemia, as studies have shown that preventing and controlling hyperlipidemia can play a major role in both primary prevention and secondary prevention of CVD^19,20^.

In our study, the main types of dyslipidemia in Shanxi Province were low HDL-C, followed by hypertriglyceridemia, findings that are consistent with those from other studies in Asian countries^18^. This phenomenon probably reflects the growing high intake of simple carbohydrates and high-fat diets that have emerged in recent decades, which clearly affect serum triglyceride concentrations^18^. In addition, the hyperlipidemia prevalence varies widely with different demographic characteristics and lifestyles. It is noteworthy that we found that the detection rate of hyperlipidemia in participants with excessive intakes of fresh vegetables was unexpectedly high, which may be related to a conscious increase of vegetable consumption among these participants upon konwing that they had hyperlipidemia. The prevalence of hyperlipidemia in the population over the age 60 years has been reduced, probably because more people over the age of 60 years with hyperlipidemia have died from hyperlipidemia complications or other diseases. As shown in Fig. 2 and Table 1, men under age 60 years were more inclined to have higher prevalence of dyslipidemia than women in the same age group (P < 0.10) whereas the detection rate of dyslipidemia increased with age in women (P < 0.10); women over 60 years old had a significantly higher detection rate than men in the same age group (P < 0.10), which may be associated with reduced sex hormones.

At the same time, hyperlipidemia was found to be closely related to hypertension and diabetes mellitus. A total 52.2% of diabetes mellitus patients and 49.1% of patients with hypertension had dyslipidemia, whereas the prevalence of dyslipidemia among their counterparts without diabetes mellitus or hypertension was 41.6%, 37.8%, respectively. Previous studied have showed that diabetes mellitus and hypertension are associated with dyslipidemia, even after adjusting for other relative variables^21,22^. Although the relevant factors of hyperlipidemia have been identified, how these related factors are associated with hyperlipidemia have rarely been studied.

The Bayesian network model shows connections between hyperlipidemia and those related factors that were established by a complex network structure. Of these, direct connections between gender, age, region, occupation, vegetable intake level, physical activity, BMI, central obesity, diabetes mellitus, and hyperlipidemia were found (Fig. 3) whereas hypertension was indirectly linked to hyperlipidemia. The BN model can also be used to figure out the interrelationship between related factors of hyperlipidemia whereas multivariate logistic regression cannot for its limitations of independency^23^. The results summarized above show that the BNs model can be used to assess the dependency of hyperlipidemia on all factors included in the model, as well as the interrelationships between these factors, which makes it convenient for exploring the internal relationships between factors, to thereby improve hyperlipidemia prevention.

BNs can infer the probability of an unknown node (hyperlipidemia) based on the state of known nodes^16^. For example, according to the reasoning model of hyperlipidemia, we know that if a person engages in insufficient exercise, the detection rate of hyperlipidemia increases from 42.6% to 45.1% (Supplementary Fig. S1) whereas the prevalence reaches 55.2% if this person has central obesity (Supplementary Fig. S2). If this person is also obese, then he has a 59.4% probability of having hyperlipidemia (Supplementary Fig. S3); the probability increases to 65.0% when this person has diabetes mellitus concurrently (Supplementary Fig. S4). Therefore, maintaining body weight within a reasonable range, getting sufficient physical exercise, and diabetes prevention should be given priority, to reduce the occurrence of hyperlipidemia.

Although they are current method of choice for evaluating factors related to dyslipidemia, multivariable logistic regression models are often constrained by issues such as an inability to find how factors impact on outcome variable, inability to assess the interrelationships between factors, and independent restrictions of variables, which contribute to the conundrum of implementation to comprehensively analyse the factors affecting dyslipidemia. However, BNs can solve this problem and have their own merits. BNs can combine prior information with sample information to avoid subjective bias by only using prior knowledge, as well as avoiding bias by only using sample knowledge^24^. BNs can also predict the probability of an unknown node by the state of known nodes^25^, deal with problems of incomplete data^26^, handle situations of uncertain information and excessive variables using probability theory with a solid mathematical basis, and can display results as an intuitive graphic^27^. However, BNs also have some limitations, for example, the direct arcs of the constructed BNs do not provide cause–effect relationships but rather show how the various parameters are influenced (statistically) by each other. Only the BNs established by causal relationship with directed edges indicate cause–effect relationships^28^.

## Methods

### Study participants

**Figure 5:**
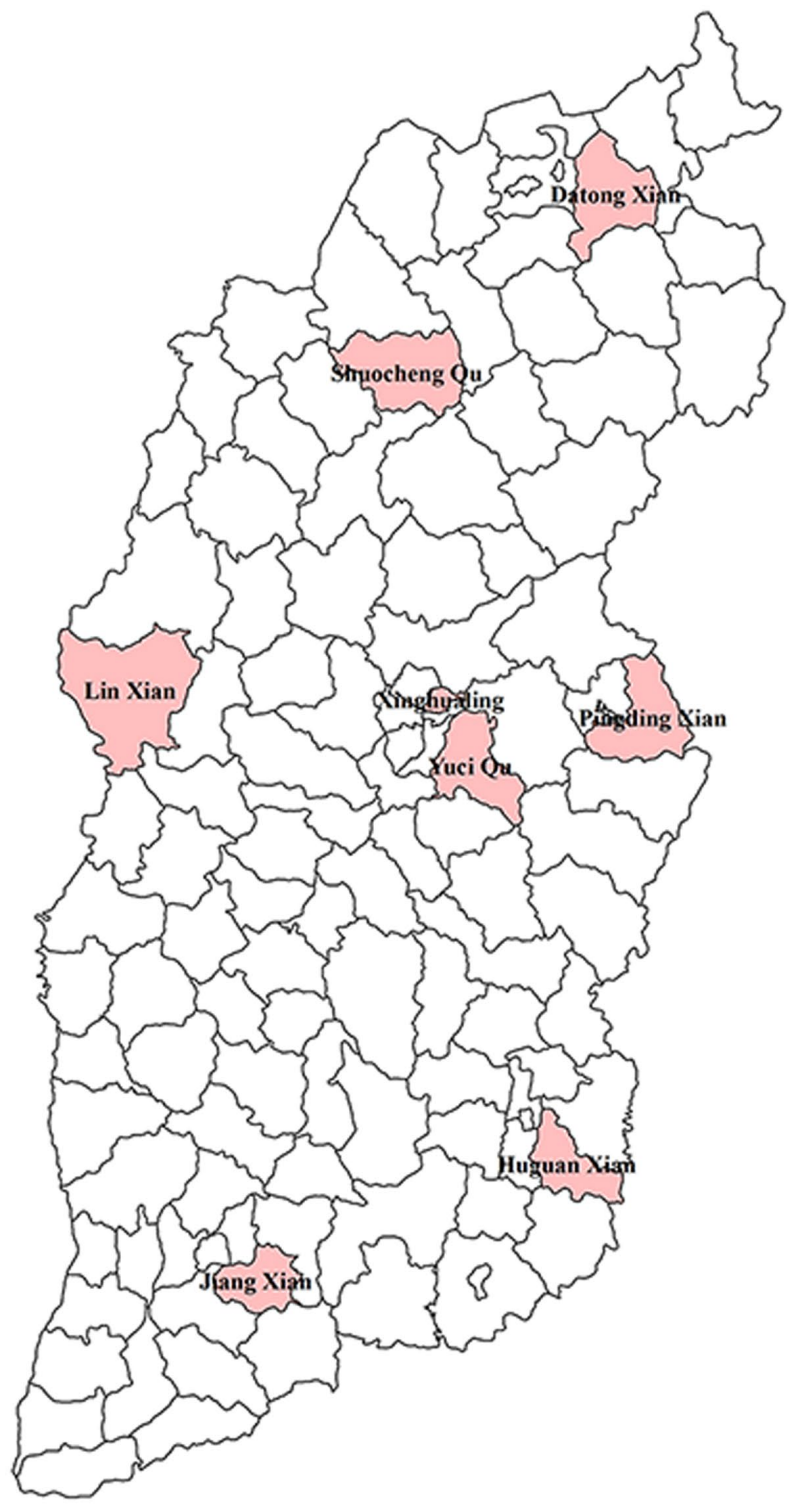
The distribution of monitoring points in Shanxi Province. The figure was plotted using ArcGIS 10.2 (www.esri.com/).

Participants were included in the China National Chronic Diseases Survey, which was conducted in Shanxi Province in 2013. In this survey, a multi-stage stratified random sampling method was used to obtain representative samples. In the first stage, eight representative monitoring points were randomly selected in Shanxi Province. Figure 5 shows the distribution of these monitoring points in the province; it can be seen that the monitoring points are evenly distributed in Shanxi Province. In the second stage, four townships or streets were randomly selected from each monitoring point. In the third stage, three villages/committees/organizations were randomly selected from each township/street. The fourth stage involved random selection of one group containing 50 households from each selected residential committee/village/organization. In the final stage, a standard Kish table was adopted to randomly select one person aged 18 years or above from each household. If the selected individual was unable or ineligible to participate, a similar household in the same or an adjacent neighbourhood or village was randomly selected as a replacement. Finally, a total of 4,776 participants completed the survey and physical examination. This study was approved by the China Chronic Disease Center Ethics Committee, with reference number 201307. Informed consent was signed by all study participants or their agents. All experiments were performed according to the relevant guidelines and regulations.

The eligibility criteria for the study was all residents aged 18 years or above who had been living in the monitoring area for more than 6 of the previous 12 months. Exclusion criteria for this study were those residents who lived in functional areas, such as sheds, military or student dormitories, nursing homes, and so on.

### Data collection

#### Questionnaire interview

A written informed consent was given to all participants before the collection of data. After signing the informed consent, all participants received a questionnaire established by the China Center for Disease Control and Prevention (CDC) on chronic disease. A direct face-to-face questionnaire interview was carried out by uniformly trained investigators. The questionnaire included information on general demographic characteristics (such as region, age, sex, level of education, and occupation), lifestyles (such as smoking, drinking, dietary habits, and physical activity), and past medical history (such as hypertension and diabetes mellitus).

### Anthropometric measures

Anthropometric measurements including height, weight, waist circumference and blood pressure were taken. Height and weight were measured with participants wearing light indoor clothing and no shoes, following a standardized protocol, to the nearest 0.1 cm and 0.1 kg, respectively. Waist circumference was measured twice at the midpoint level of the midaxillary line between the lower edge of the ribcage and the iliac crest. After ensuring that the difference between the two measurements was less than 2 cm, the second measured value was then recorded. Blood pressure were measured while participants were in a seated position following a 5-min rest period. Third consecutive blood pressure (BP) readings were taken using an electronic sphygmomanometer (OMRON HEM-7071 or HEM-770A), with an accuracy of 1 mmHg; the averages were then calculated for a final blood pressure reading.

### Laboratory assays

Laboratory assays included blood glucose, blood lipids, glycosylated haemoglobin, and so on. Samples for detecting blood sugar were kept in cold storage at 2–8 °C until they were sent to a local laboratory for measurement of fasting glucose and 2-h postprandial plasma glucose in 48 hours. Other samples were stored at −60 to −80 °C in specialized areas, or at −20 °C in areas that did not have cryopreservation facilities, until being transported to the medical inspection organizations designated by the state for blood lipid and glycosylated haemoglobin testing within one month.

### Bayesian networks (BNs)

A Bayesian network is a directed acyclic graph (DAG) based on probability theory and graph theory^29^, which consists of nodes representing the variables U = {X_i_, …, X_n_} and directed edges symbolizng the relationships between the variables^13,27,30,31^. If there is an edge from X_i_ to X_j_, then we say that node ${{\rm{X}}}_{{\rm{i}}}$ is the parent of X_j_ and X_j_ is the child of X_i_. Each node has a conditional probability distribution table (CPT), which quantitatively describes the probability dependence of the nodes and its parent nodes^28^. From the perspective of probability theory, a BN represents the joint distribution of a set of random variables, according to the chain rule and conditional independence, the joint distribution of a series of random variables X = {${X}_{1},\cdots ,{X}_{n}$ can be written as:

1 $$P({X}_{1},\ldots ,{X}_{n})=P({X}_{1})P({X}_{2}|{X}_{1})\ldots P({X}_{n}|{X}_{1},{X}_{2},\ldots ,{X}_{n-1})={{\rm{II}}}_{1}^{n}P({X}_{i}|\pi ({X}_{i}).$$

$\pi ({X}_{i})$ is the collection of the parent of *X*_i_,$\,\pi ({X}_{i})$ *⊆* $\{{X}_{1},\cdots ,{X}_{i-1}\}$, given the value of $\,\pi ({X}_{i});\,{X}_{i}$ is conditionally independent of other variables^32^ in $\{{X}_{1},\cdots ,{X}_{i-1}\}$.

### Tabu search algorithm

A tabu search (TS) algorithm starts from a feasible initial solution; the neighbour solutions are generated through a sequence of moves. If a movement in a certain direction is found that makes the objective function value change the most, it will be placed into the tabu list and is considered one of the optimal solutions in the local area, unless it was in the tabu list. Then the initial solution is replaced with this new optimal solution and it continues to move to its neighbourhood, looking for the next optimal solution nearest the previous optimal solution, repeating the cycle continuously until the convergence criteria are met, at which point the search process is stopped^33^.

### Definitions

In accordance with Chinese Guidelines on Prevention and Treatment of Dyslipidemia in Adults published in 2007, hyperlipidemia was defined as the presence of any one of the following four conditions: hypercholesterolemia (total cholesterol (TC; ≥ 6.22 mmol/L); hypertriglyceridemia (triglycerides (TG) ≤ 2.26 mmol/L); low levels of high-density lipoprotein cholesterol (HDL-C; < 1.04 mmol/L); or high levels of low-density lipoprotein cholesterol (LDL-C; ≥ 4.14 mmol/L)^9^.

According to Guidance on Prevention and Control of Hypertension in Chinese Residents, hypertension was defined as individuals with an average measured systolic blood pressure ≥ 140 mmHg and/or diastolic blood pressure ≥ 90 mmHg, or who reported having been diagnosed with hypertension or receiving BP-lowering treatment^34^. Diabetes mellitus was defined as fasting glucose ≥ 7.0 mmol/L or 2-h postprandial plasma glucose ≥ 11.1 mmol/L or who reported having been diagnosed with diabetes mellitus^35^.

Participants who reported smoking ≥ 1 cigarette a day for the previous 6 months were defined as smokers. Body weight was categorized as normal weight (body mass index (BMI) ≥ 18.5 kg/m^2^ and < 24 kg/m^2^), overweight (BMI ≥ 24 kg/m^2^ and < 28 kg/m^2^), and obese (BMI ≥ 28 kg/m^2^)^36^. Central obesity refers to waist circumference ≥ 85 cm for male and ≥ 80 cm for female^37^. Bradycardia means heart rate less than 60 beats/min, tachycardia refers to heart rate greater than 100 beats/min, and normal heart rate is between 60 and 100 beats/min^38^. Physical activity was categorized into low, moderate, and high groups based on the upper quartile and lower quartile of weekly metabolic equivalent.

### Statistical analyses

Categorical variables were summarized as proportions, and chi-square tests were applied to compare categorical variables. Multivariate logistic regression and Bayesian networks were used to explore the factors related to hyperlipidemia.

Statistical description, chi-square tests, and multivariate logistic regression were performed using IBM SPSS Version 22 (IBM Corp., Armonk, NY, USA). Significance for all statistical tests was a priori at P* < *0.10 and all P values were two-tailed; Weka 3.8.0 (Waikato Environment for Knowledge Analysis; the University of Waikato, New Zealand) was used for structural learning and parametric learning of BNs. The BN models and reasoning models were drawn using Netica (Norsys Software Corp., Vancouver, BC, Canada). In addition, the maximum likelihood method was used to obtain the values for CPT.

### Data availability

All data generated or analyzed during this study are included in its Supplementary Information files.

## Electronic supplementary material

Supplementary information

Dataset 1
