# Supplementary material for: Prevalence of hyperlipidemia in Shanxi Province, China and application of Bayesian networks to analyse its related factors
Source: Sci Rep. 2018 Feb 28;8:3750. doi: 10.1038/s41598-018-22167-2 (PMC5830606; doi:10.1038/s41598-018-22167-2)
Supplement: Supplementary file 1 — Supplementary information [file 41598_2018_22167_MOESM1_ESM.pdf]

Prevalence of hyperlipidemia in Shanxi Province, China and application of Bayesian networks to analyse its related factors

Jinhua Pan[1+], Zeping Ren[2+], Wenhan Li[1], Zhen Wei[1], Huaxiang Rao[3], Hao Ren[1], Zhuang Zhang[1], Weimei Song[1], Yuling He[2], Chenglian Li[2], Xiaojuan Yang[2], LiMin Chen[2*] & Lixia Qiu[1,*]

[1] Department of Health Statistics, School of Public Health, Shanxi Medical University, No.5 6 XinJian South Road, Taiyuan, Shanxi 030001, China

[2] Shanxi Centre for Disease Control and Prevention, Taiyuan, Shanxi 030012, China

[3] Institute for Communicable Disease Control and Prevention, Qinghai Provincial Center for Disease Control and Prevention, Xining, Qinghai 810007, China

[+] These authors contributed equally to this work

*Correspondence authors:

L.X.Q. (E-mail: qlx_1126@163.com; Tel: +86-13453189351) or

L.M.C. (E-mail: sxchenlimin@163.com; Tel: +86-13934519150)

Factors	Assignments
Gender( )	Male*=1;Female=2
Age( )	<30*=1;30~=2;45~=3;60~=4;75~=5
Region( )	Countryside*=1;City =2
Ethnic ( )	Han nationality*=1;Minority=2
Cultural level( )	Under high school*=1, High school and over=2
Occupation( )	Farmer=1;Unemployer or retirees =2;Employer =3;Others*=4
Marital status ( )	Unmarried*=1; Married or cohabiting=2;Widowhood; Divorce, or separation =3
Smoking( )	NO*=0;YES=1
Drinking status( )	Never*=1;Current=2;Former=3
Physical activity(x10)<br>Fresh fruit ( )	Low*=1;Moderate =2;High =3<br><100g/d*=1;100~200g/d=2;>200g/d=3
Vegetables ( )	<400g/d*=1;400~500g/d=2;>500g/d=3
Livestock, poultry meat ( )	<50g/d*=1;50~100g/d=2;>100g/d=3
Heat rate( )	Bradycardia* =1;Normal=2;Tachycardia =3
BMI( )	<18.5*=1;18.5~=2, 24.0~=3;28.0~=4
Central obsity( )	NO*=0;YES=1
Hypertension( )	NO*=0;YES=1
Diabetes ( )	NO*=0;YES=1
hyperlipidemia (y)	NO*=0; YES=1

* Reference standard

Supplementary Table S1. Factors and assignments

Factors	cases	hyperlipidemia	prevalence (%)		*P*
Gender					
Male	1748	798	45.7	11.735	0.001
Famale	2357	950	40.3		
Age					
<40	748	298	39.8		
40~	2301	1012	44.0	4.670	0.097
60~	1056	438	41.5		
Region					
Countryside	2581	1140	44.2	7.159	0.008
City	1524	608	39.9		
Ethnic					
Han nationality	4095	1745	42.6	0.649	0.532
Minority	10	3	30.0		
Cultural level					
Under high school	3245	1356	41.8	4.002	0.048
High school and above	860	392	45.6		
Marital status					
Unmarried	182	72	39.6		
Married or cohabiting	3501	1498	42.8	0.768	0.681
Widowhood, divorce, or separation	442	178	42.2		
Occupation					
Farmer	1976	794	40.2		
Retirees or unemployers	297	133	44.8	10.205	0.017
Employers	640	298	46.6		
Others	1192	523	43.9		

Supplementary Table S2. Comparison of differences in prevalence among different demographic characteristics

Factors	cases	hyperlipidemia	prevalence (%)		*P*
Smoking					
NO	3241	1342	41.4	8.996	0.011
YES	800	378	47.3		
Drinking status					
Never	3230	1356	42.0		
Current	634	286	45.1	2.380	0.304
Former	241	106	44.2		
Physical activity					
low	1027	464	45.2		
moderate	2052	881	42.9	7.518	0023
high	1026	403	39.3		

Supplementary Table S3. Comparison of differences in prevalence among different lifestyle

Factors	cases	hyperlipidemia	prevalence (%)		*P*
Fresh fruit					
<100g/d	2457	1043	42.5		
100-200g/d	1130	498	44.1	2.497	0.287
>200g/d	518	207	40.0		
Vegetables					
<400g/d	2578	1073	41.6		
400-500g/d	864	346	40.0	16.688	<0.001
>500g/d	663	329	49.6		
Livestock, poultry meat					
<50g/d	3650	1563	42.8		
50-100g/d	139	58	41.7	0.867	0.648
>100g/d	316	127	40.2		

Supplementary Table S4. Comparison of differences in prevalence among different intake level of dietary factors

Factors	cases	hyperlipidemia	prevalence (%)		*P*
BMI(kg/)					
<18.5	64	8	12.5		
18.5~	1533	469	30.6		
24.0~	1669	792	47.5	202.246	<0.001
28.0~	839	479	57.1		
Heart rate					
Bradycardia	139	51	36.7		
normal	3878	1661	42.8	2.173	0.337
Tachycardia	88	36	40.9		
Central obesity					
NO	1369	385	28.1	175.644	<0.001
YES	2736	1363	49.8		
Hypertension					
NO	2370	896	37.8	52.321	<0.001
YES	1735	852	49.8		
Diabetes mellitus					
NO	3720	1547	41.6	16.099	<0.001
YES	385	201	52.2		

Supplementary Table S5. Comparison of differences in prevalence among different physical condition

Parent nodes		hyperlipidemia	
BMI	Central Obesity	NO	YES
<18.5	YES	55	45
<18.5	NO	91.964	8.036
18.5~	YES	60.925	39.075
18.5~	NO	73.564	26.436
24.0~	YES	50.681	49.319
24.0~	NO	61.913	38.087
28.0~	YES	42.693	57.309
28.0~	NO	57.692	42.308

Supplementary Table S6. the conditional probability table of hyperlipidemia

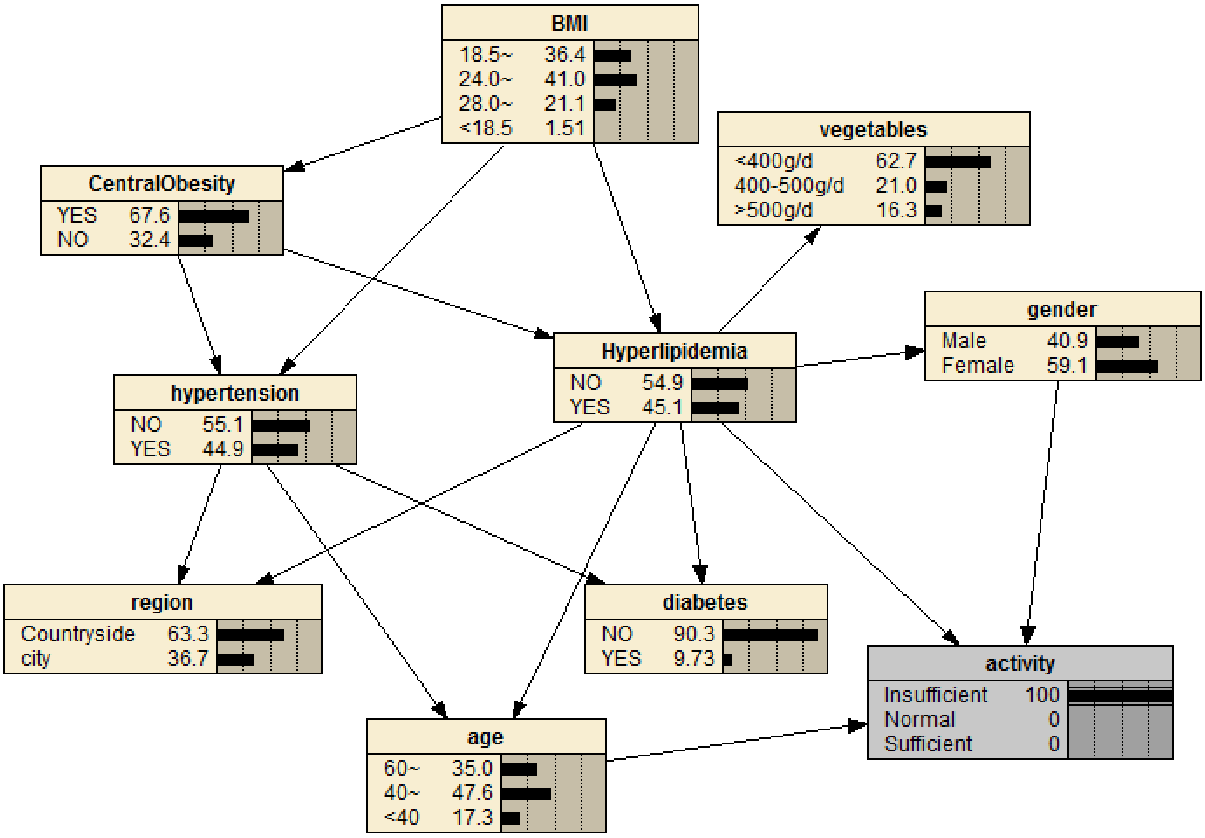

Supplementary Figure S1. Bayesian network I under known evidence variables

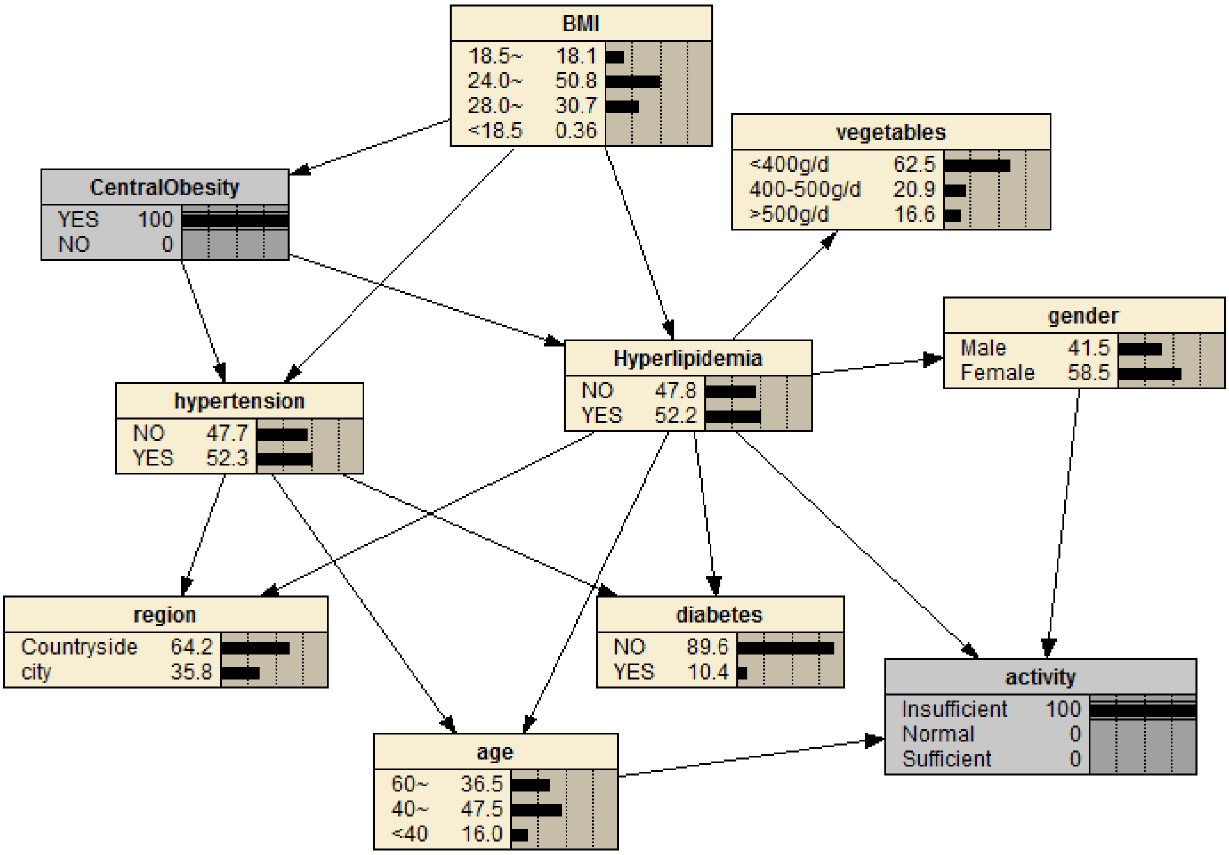

Supplementary Figure S2. Bayesian network II under known evidence variables

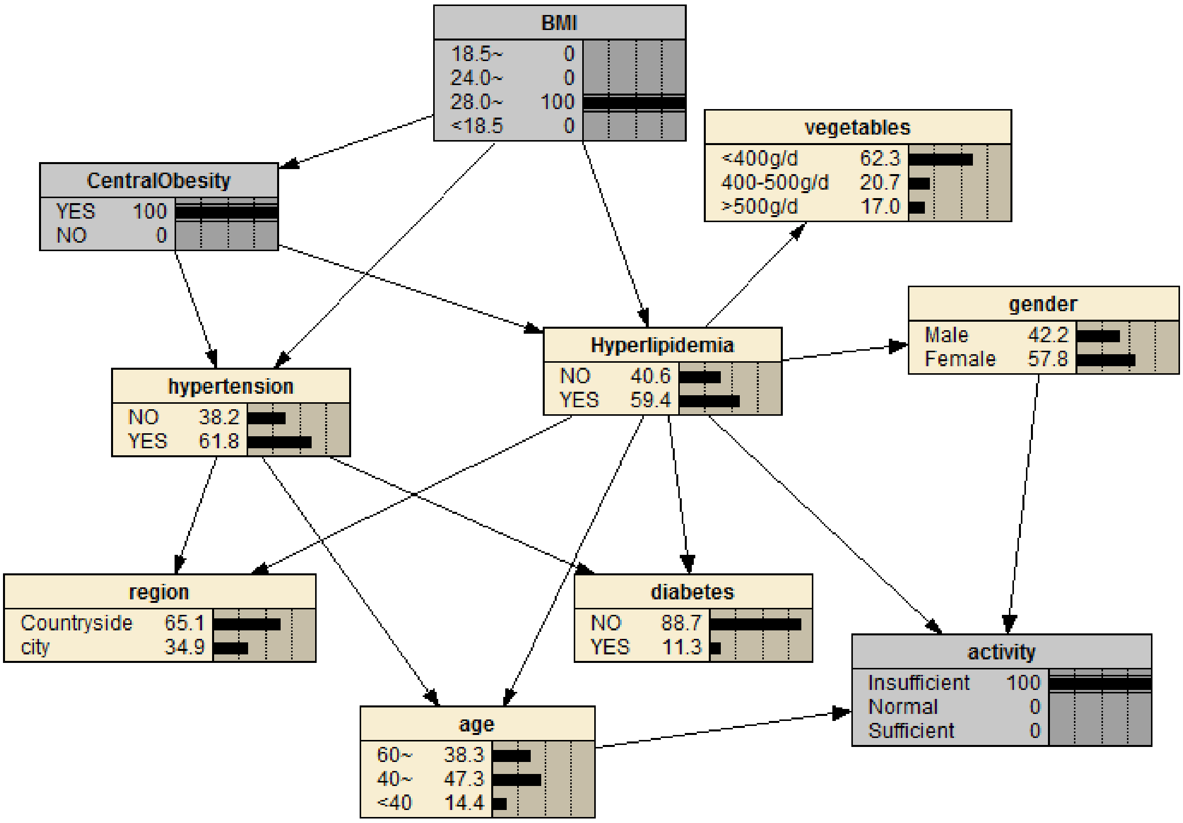

Supplementary Figure S3. Bayesian network III under known evidence variables

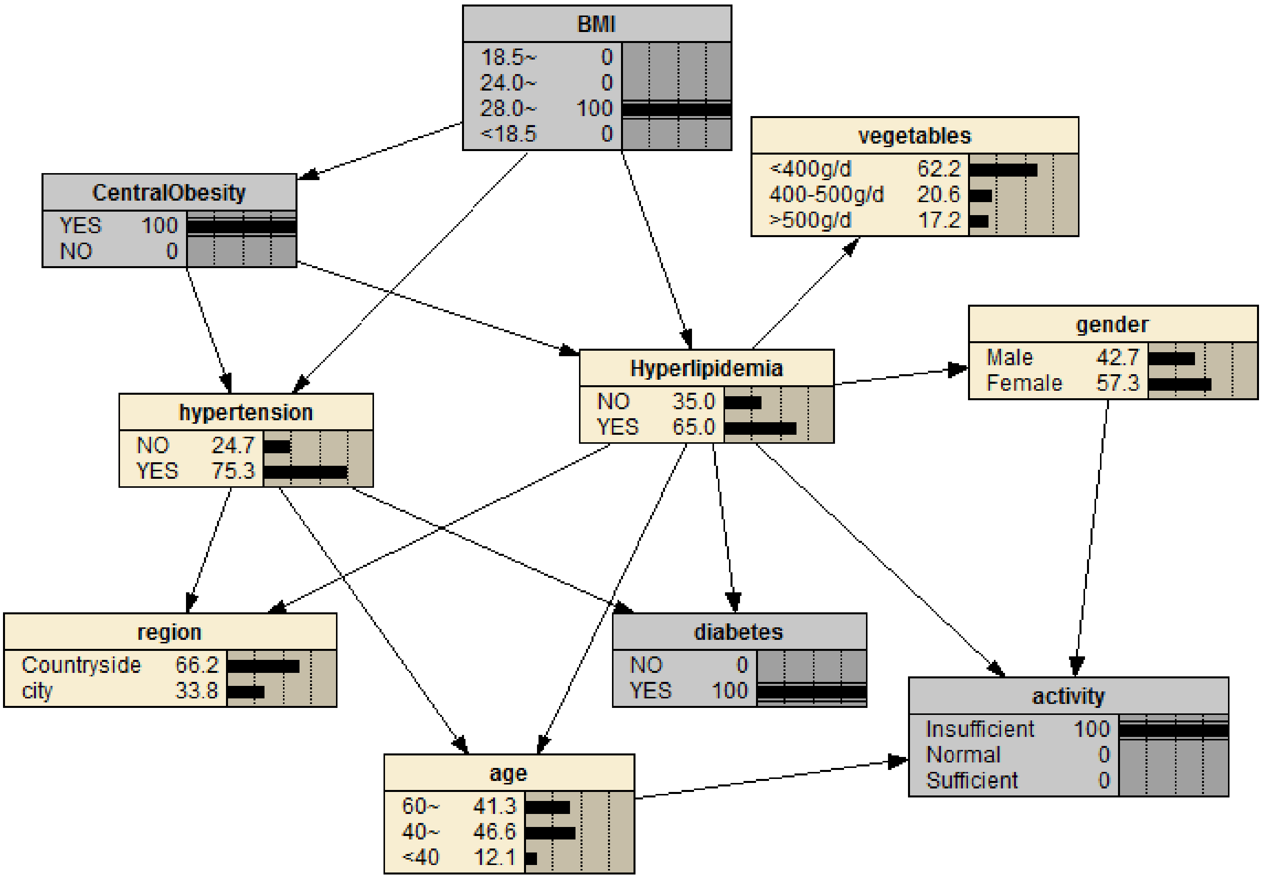

Supplementary Figure S4. Bayesian network IV under known evidence variables

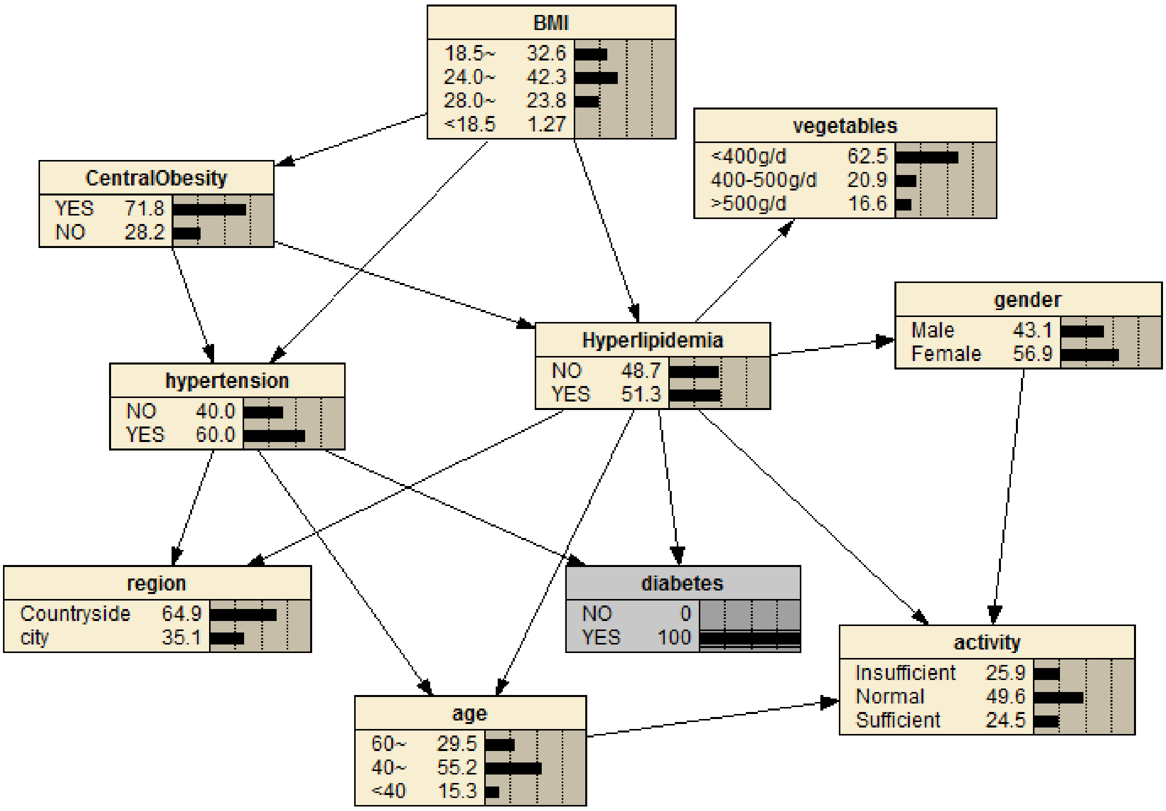

Supplementary Figure S5. Bayesian network V under known evidence variable

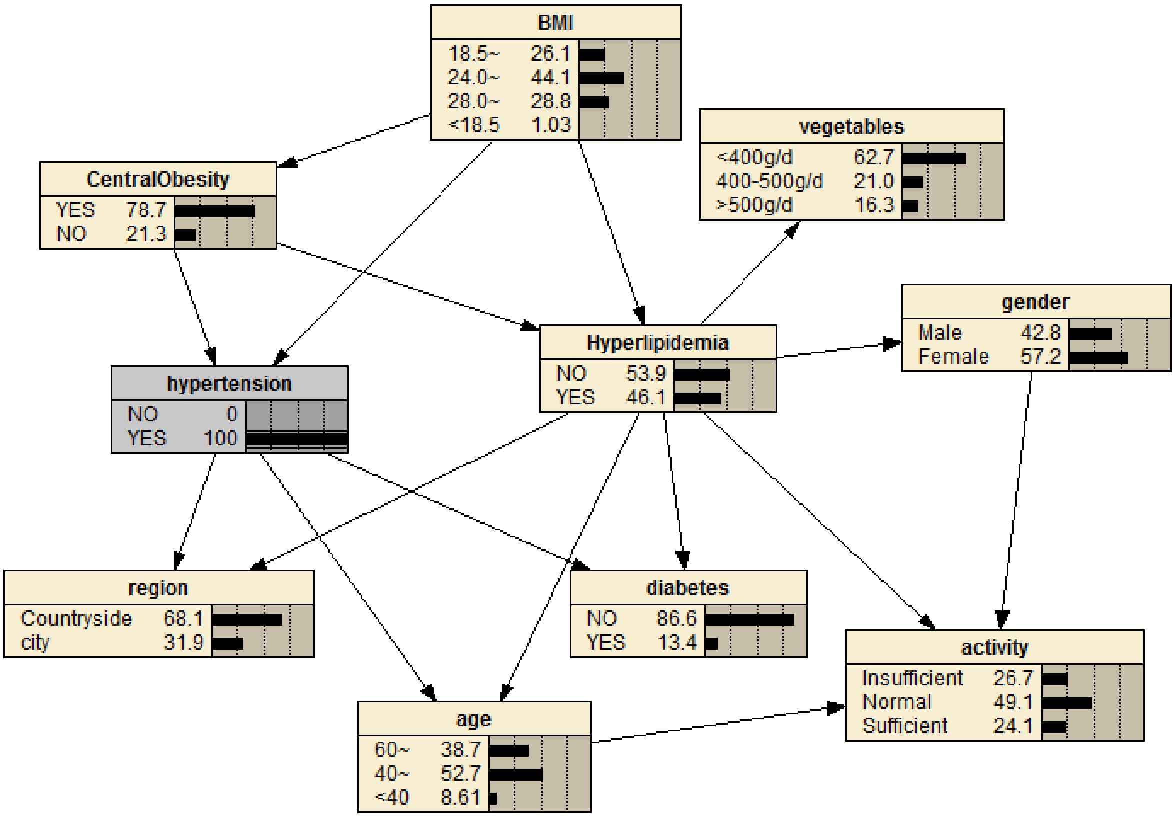

Supplementary Figure S6. Bayesian network VI under known evidence variable

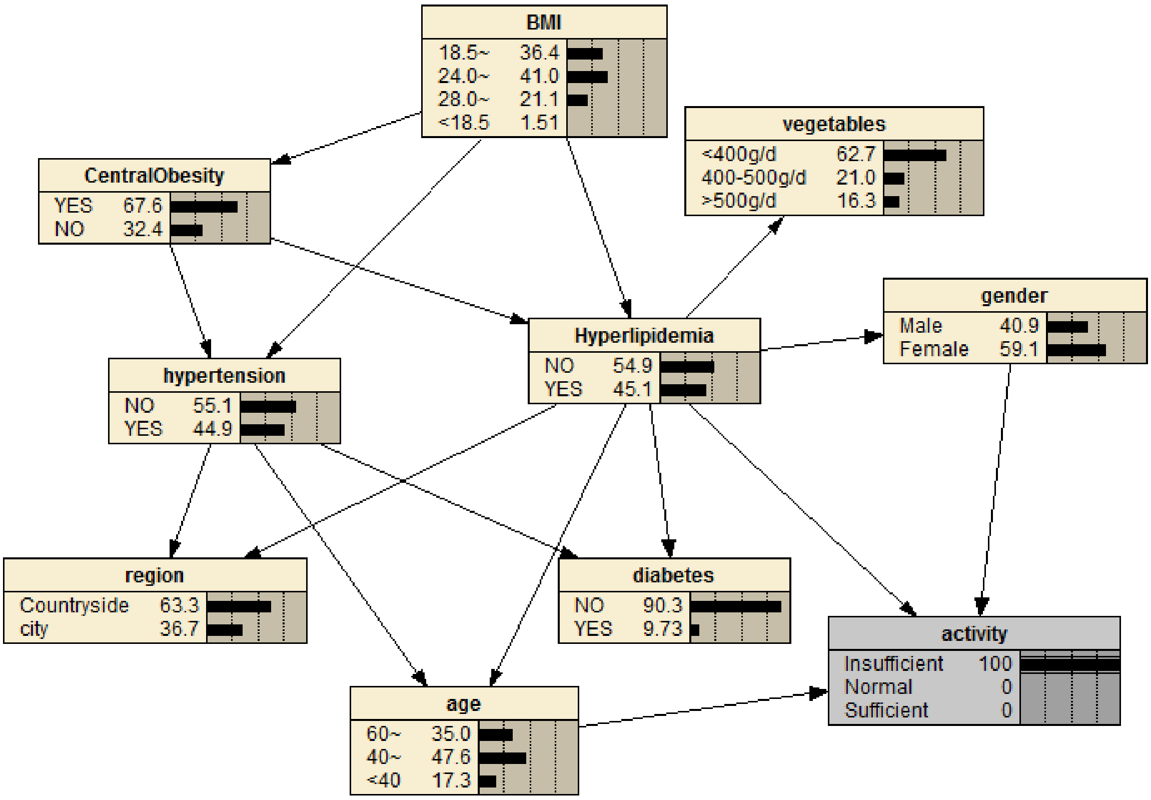

Supplementary Figure S7. Bayesian network VII under known evidence variable
